# Exploratory Profiling of Urine MicroRNAs in the *dy^2J^/dy^2J^* Mouse Model of LAMA2-CMD: Relation to Disease Progression

**DOI:** 10.1371/currents.md.d0c203c018bc024f2f4c9791ecb05f88

**Published:** 2018-08-27

**Authors:** Bernardo Moreira Soares Oliveira, Kinga I Gawlik, Madeleine Durbeej, Johan Holmberg

**Affiliations:** PhD Student, Experimental Medical Science, Lund University, Lund, Sweden; Muscle Biology Unit, Lund University, Lund, Sweden; Integrative Biology and Physiology, University of California, Los Angeles, California, United States

## Abstract

Circulating microRNAs (miRNAs) are being considered as non-invasive biomarkers for disease progression and clinical trials. Congenital muscular dystrophy with deficiency of laminin α2 chain (LAMA2-CMD) is a very severe form of muscular dystrophy, for which no treatment is available. In order to identify LAMA2-CMD biomarkers we have profiled miRNAs in urine from the *dy^2J^*/*dy^2J^* mouse model of LAMA2-CMD at three distinct time points (representing asymptomatic, initial and established disease). We demonstrate that unique groups of miRNAs are differentially expressed at each time point. We suggest that urine miRNAs can be sensitive biomarkers for different stages of LAMA2-CMD.

## Introduction

Laminin α2 chain-deficient congenital muscular dystrophy, or LAMA2-CMD, is a severe form of muscular dystrophy caused by mutations in the *LAMA2* gene. Genotype-phenotype analyses have demonstrated that complete deficiency of laminin α2 chain leads to a more severe phenotype whereas partial absence leads to a milder disease course. The clinical manifestations of complete laminin α2 chain-deficiency include profound hypotonia at birth, widespread muscle weakness, proximal joint contractures, scoliosis and delayed motor milestones. Patients may achieve unsupported sitting but very few children acquire independent ambulation. Individuals with partial deficiency often have later onset of proximal muscle weakness and delayed motor milestones but achieve independent ambulation 1.

There are several mouse models for laminin α2 chain-deficiency that adequately represent the clinical heterogeneity of LAMA2-CMD and phenocopy the skeletal muscle changes. The *dy*^*3K*^*/dy*^*3K*^ mouse completely lacks laminin α2 chain and displays a very severe muscular dystrophy with a median survival of three weeks whilst the *dy*^*2J*^*/dy*^*2J*^ mouse model has slightly reduced expression and shows a relatively mild muscular dystrophy with a life span of several months. In both models, skeletal muscle is characterized by repeated cycles of degeneration/regeneration and pathological fibrosis. Consequently, *dy*^*3K*^*/dy*^*3K*^ and *dy*^*2J*^*/dy*^*2J*^ mice weigh less and display impaired skeletal muscle function 2. Diagnosis of LAMA2-CMD and knowledge of underlying pathogenetic mechanisms have greatly improved due to advances in clinical and pre-clinical studies involving LAMA2-CMD patient material and the above mentioned mouse models (as well as other animal models). However, early detection, assessment of disease progression and response to treatment are still major challenges. Hence, it would be important to find novel biomarkers that could facilitate diagnosis and prognosis and aid in evaluating preclinical as well as clinical trial results. The traditional biomarker for muscular dystrophy is creatine kinase (CK), coupled with histological inspection of muscle biopsies. Unfortunately, CK is not very reliable as it is sensitive to age, sex, physical exertion, stress and diet 3, and muscle biopsies are invasive. Therefore, there is an urgent need for more reliable and less invasive biomarkers for LAMA2-CMD and muscular dystrophies in general.

After their discovery in the early nineties, microRNAs (also called miRNAs or miRs) have been extensively studied for their biological roles and biomarker potential. miRNAs are short (18-24 nt) non-coding RNAs that post-transcriptionally regulate protein synthesis by complementary-binding messenger RNA, which leads to degradation of the latter or translation inhibition 4. Their presence in extracellular fluids, such as blood and urine, along with miRNA dysregulation in various diseases, including muscular dystrophy 5, has spurred extensive biomarker research 6^,^^7^^,^^8^^,^^9^^,^^10^. Indeed, we have previously demonstrated that laminin α2 chain-deficiency is associated with miRNA dysregulation in skeletal muscle and plasma 11. In this study we aimed at profiling miRNA expression in urine from *dy*^*2J*^*/dy*^*2J*^ mice to assess their potential for monitoring disease progression. Three distinct time points (three, four and six weeks of age) were chosen to represent asymptomatic, initial symptoms and established disease, respectively. Here we show that distinct sets of miRNA characterise each time point whilst CK fails to differentiate between them.

## Materials and Methods

**Ethics Statement**

Wild-type and *dy*^*2J*^*/dy*^*2J *^(B6.WK-*Lama2dy-2J/*J) mice were purchased from Jackson laboratory and bred in the Biomedical Center according to institutional animal care guidelines. Permission was given by the Malmö/Lund (Sweden) ethical committee for animal research (ethical permit number M152-14).

**Tissue collection**

Three-, four- and six-week-old control and *dy*^*2J*^*/dy*^*2J*^ mice (n = 5 per group) were sacrificed by cervical dislocation. Quadriceps muscles were dissected for histology and embedded in paraffin.

**Histology and morphometric analyses**

Muscle sections were stained with haematoxylin & eosin 12 or picro sirius red/fast green 11. Stained cross-sections were scanned using Aperio’s Scanscope CS2 (with Scanscope console v. 8.2.0.1263) and representative images were created using Aperio software.

Centrally nucleated muscle fibres representing regenerating muscle cells and peripherally nucleated non-regenerating muscle cells were counted in quadriceps femoris using ImageJ software version 1.45i (NIH, Bethesda, MD). The whole area of each muscle cross section was considered.

**Creatine kinase assay**

Blood was collected from heart puncture and transferred to anticoagulant tubes (EDTA) and centrifuged at 1100 × g for 10 min. at 4°C. Plasma was analysed at Clinical Chemistry Laboratory at Skåne University Hospital. The CK_P_S Cobas method was used to quantify enzyme activity.

**Fast green/sirius red quantification**

Collagen content was quantified by a colorimetric method as described 13. Briefly, approximately 15 paraffin sections of 15 µm were placed in a plastic tube. Paraffin removal was accomplished by immersing the sections in the following solutions: 5 min. xylene, 5 min. xylene/ethanol (1:1), 5 min. ethanol, 5 min. ethanol/water (1:1), 5 min. water. The sections were then stained with fast green/sirius red for 30 min. (rotating). The tissue was washed with distilled water until excess dye was removed and the solution was clear. One ml of 0.1 N NaOH was added to elute colours. The eluted fraction was analysed at 560 nm and 605 nm to estimate total protein content (fast green) and collagen content (Sirius red), respectively.

**Grip strength**

Forelimb grip strength was measured on a grip-strength meter (Columbus Instruments, Columbus, OH) as previously described 14. In short, the mouse was held by the base of the tail and allowed to grasp the flat wire mesh of the pull bar with its forepaws. When the mouse got a good grip it was slowly pulled away by its tail until it released the pull bar. Each mouse was allowed to pull the pull bar five times. The two lowest values were rejected and the mean of the three remaining values was counted. Animals were not subjected to any training prior to the experiment.

**Urine collection**

Individual mice were manually handled on top of a grid placed above a collection plate. The mouse was grabbed by the neck and tail and placed in an upright position. When stressed by the handling the mouse would urinate onto the plate. The urine was pipetted into a tube and stored at -80°C.

**Isolation of RNA and RNA sequencing**

Total RNA from urine was extracted with Qiagen miRNeasy Mini Kit following the manufacturer’s instructions. One hundred and twenty microliters of urine were pooled from two or three animals into one sample.

Total RNA isolated from urine was sent to the Uppsala Genome Centre for high-throughput sequencing on the IonTorrent platform (ThermoFisher Scientific). The raw sequencing data is deposited in the European Nucleotide Archive under accession number PRJEB23307.

**Bioinformatics analyses**

Bioinformatics analyses were performed in conjunction with the Bioinformatics Long-term Support (WABI – SciLifeLab). Shortly, raw reads between 18 and 24 nt were kept for further analyses. These were mapped to the mouse hairpin miRNA sequences (miRBase v. 22) using bowtie 15 (v. 1.2, Johns Hopkins University). Read counts were calculated with HTSeq 16 and miRBase annotation (v. 21). Differential expression analysis was performed in the R statistical environment (v. 3.3.2, R Foundation for Statistical Computing) with the Bioconductor package DESeq2 17 (v. 1.12.3), with significance set at adjusted p lower than 5%.

**Statistical analysis**

The statistical analysis of next-generation sequencing data was done as described above. The remaining analyses were performed in the IPython 18 environment using the SciPy (v. 0.18.1) 19 and statsmodels packages (v. 0.61). Difference between groups was assessed by one-way analysis of variance. Significance was set at the 5% level. Data are presented as mean ± SEM.

## Results

**Characterisation of disease stages**

**Figure:**
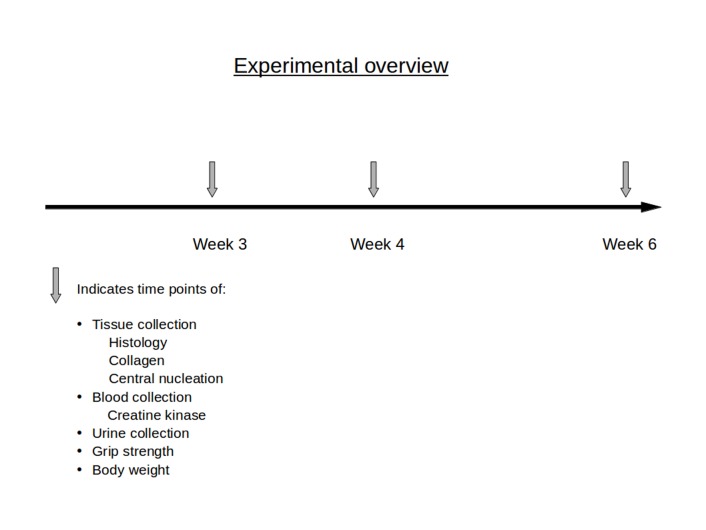
**Fig. 1:** Experimental overview. The arrows (upper part) indicate the different time points when analyses (lower left) were performed.

In order to profile miRNA expression in urine from *dy*^*2J*^*/dy*^*2J*^ mice at asymptomatic, initial and established stages of the disease, we first assessed body weight, muscle function, muscle histology, and creatine kinase levels in three-, four- and six-week-old *dy*^*2J*^*/dy*^*2J*^ and wild-type (WT) littermate mice (Figure 1). Independent of age, *dy*^*2J*^*/dy*^*2J*^ male mice weighed less than wild-type mice. This reduction in body weight was only significant at six weeks of age among female *dy*^*2J*^*/dy*^*2J*^ mice (Figure 2A). Grip strength to body weight ratio is an indicator of muscle function that allows comparison of animals with different body weights. It is also an indicator of muscle mass change as skeletal muscle is the main tissue that produces force. At three weeks of age there was no difference in normalised grip strength, indicating no functional decline at this age, which is in accordance with the lack of overt symptoms (Figure 2B). At four and six weeks of age the *dy*^*2J*^*/dy*^*2J*^ groups had lower normalised grip strength (Figure 2B). A similar result was found by McKee et al. (2017), where *dy*^*2J*^*/dy*^*2J*^ mice had similar normalised forelimb grip-strength to wild-type mice at 3 weeks of age, with a sharp decline thereafter 20.

**Figure:**
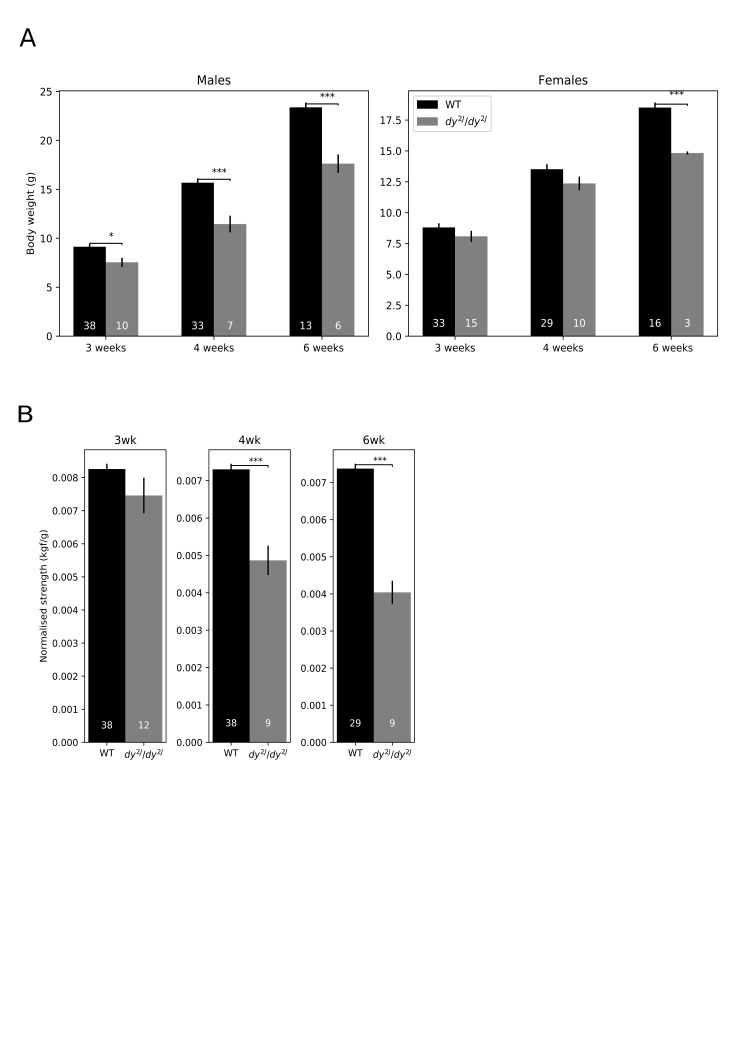
**Fig. 2:** Male dy /dy mice weight less than WT and display impaired muscle function. A: Body weight for males and females WT and dy /dy ; B: Normalised grip strength at indicated time points. * p < 0.05, ** p < 0.01, *** p < 0.001.

In order to assess if impaired muscle function was reflected by abnormal histology, we inspected haematoxylin-eosin and picro-sirius red/fast green stained sections. We found that the first visual signs of muscle pathology appeared at four of age weeks and two weeks later the diseased phenotype was evident (Figure 3A). The only indication of disease at 3 weeks of age was a low degree of inflammation (data not shown). Central nucleation was measured as an index of muscle regeneration. At three weeks of age we did not observe any signs of regeneration in *dy*^*2J*^*/dy*^*2J*^ muscle. However, at subsequent time points there was a dramatic increase in the number of regenerating fibres in *dy*^*2J*^*/dy*^*2J*^ muscle (Figure 3B).****A hallmark of muscular dystrophies is the progressive replacement of skeletal muscle by fibrous tissue. At three and four weeks of age, there was no difference in collagen content (assessed by picro-sirius red absorbance) between *dy*^*2J*^*/dy*^*2J*^ and wild-type muscle (Figure 3, C and D). However, at six weeks of age there was a significant increase in collagen content in *dy*^*2J*^*/dy*^*2J*^ muscle (Figure 3, C and D).

CK, a classical biomarker for skeletal muscle disease, was elevated in *dy2J/dy2J* serum at all time points (Figure 3E). Furthermore, CK levels in *dy2J/dy2J* serum did not differ between three and six weeks of age (not shown).

**Figure:**
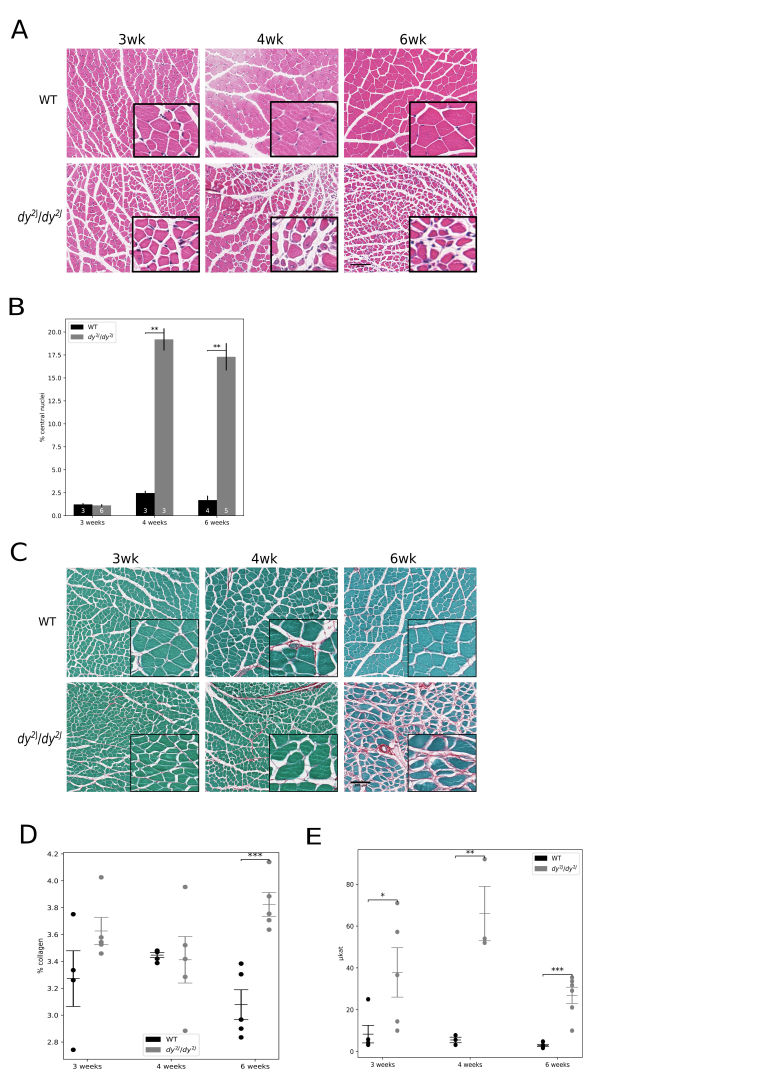
**Fig. 3:** Progressive muscle deterioration and collagen accumulation as evidenced by histology and CK concentration. A: Haematoxylin and eosin staining; B: Central nucleation quantification (as percentage of fibres with central nuclei); C: Fast green/sirius red staining: collagen is coloured red; D: Quantification of collagen as percentage of total protein; E: CK. Scale bars = 100 μm; * p < 0.05, ** p < 0.01, *** p < 0.001. Source: Fig. 3C originally published in: Moreira Soares Oliveira B, Durbeej M, Holmberg J (2017) Absence of microRNA-21 does not reduce muscular dystrophy in mouse models of LAMA2-CMD. PLoS ONE 12(8): e0181950. https://doi.org/10.1371/journal.pone.0181950.

In summary, these data revealed that there are no signs of pathology in three-week-old *dy*^*2J*^*/dy*^*2J*^ mice, but subsequently a gradual disease progression occurs. Hence, we decided to profile urine miRNAs in three-, four- and six-week-old animals.

**MiRNA profiling** **in urine**

We detected more than 700 miRNAs in mouse urine at each time point: 773 at three weeks, 764 at four weeks and 703 at six weeks of age. Among these, the number of differentially expressed miRNAs also varied. Five, 18 and 17 miRNAs, respectively, were differentially expressed at three, four and six weeks of age (Table 1, supplemental figures). We also found that distinct miRNA profiles were associated with each time point, i.e. there was minimal overlap between differentially expressed miRNAs at three, four and six weeks of age. Only miR-1957a and miR-675 were differentially expressed both at three and six weeks of age, and miR-181 was differentially expressed at four and six weeks of age. However, miRNA-181 was down-regulated at four weeks but up-regulated at six weeks of age (Table 1). Furthermore, we found that myomiRs (miR-1, miR-133 and miR-206) and muscle-enriched miRNAs (miR-181a and miR-486) dominate the differentially expressed miRNA panel at six weeks of age.

## Discussion

For the past couple of decades miRNAs have been extensively studied for their biomarker potential. However, most investigations use biopsies or blood samples for this purpose, which are invasive. CK assessment (along with morphological analysis) is often part of the standard diagnostic protocols for muscle wasting diseases. The reliability of this method has long been questioned as CK is responsive to age, sex, stress, physical exertion and diet 3. It is largely a binary analysis, indicating recent muscle damage but not cause or severity. Here, we show that CK measurement cannot be used to distinguish disease severity as it was elevated in *dy*^*2J*^*/dy*^*2J*^ mice already from three weeks onwards. Furthermore, CK levels did not differ between three- and six-week-old *dy*^*2J*^*/dy*^*2J*^ mice. We also observed higher variability in the diseased group compared to controls, making it difficult to establish a cut-off value. Considering the aforementioned limitations, we have opted for a less invasive intervention and profiled miRNAs in urine from a LAMA2-CMD mouse model at three distinct time points (asymptomatic, initial symptoms and established disease). We found that distinct miRNA profiles are associated with each time point. Specifically, we demonstrated that: 1) Some miRNAs are differentially expressed in urine from *dy*^*2J*^*/dy*^*2J*^ mice at three weeks of age although skeletal muscles appear histologically and functionally normal at that age with no obvious signs of muscle regeneration or fibrosis; 2) The highest number of differentially expressed miRNAs is seen in urine from four-week-old *dy*^*2J*^*/dy*^*2J*^ mice, a time point when these mice display dystrophic characteristics including increased myofibre regeneration (but no fibrosis) and functional decline, and finally: 3) Differentially expressed miRNAs in urine from six-week-old animals are predominantly myomiRs (i.e. miRNAs that are specific for or enriched in skeletal muscle) corresponding to fully developed muscle pathology with a high degree of muscle fibre regeneration and fibrosis. Thus, we suggest that urine miRNAs can be sensitive biomarkers for different stages of LAMA2-CMD.

Our analysis showed that at three weeks of age the miRNA with the largest fold change is miR-675-3p, followed closely by its -5p counterpart. Both miRNA are derived from exon 1 of the long non-coding RNA H19. H19 is highly expressed during embryonic phases but strongly repressed after birth, with significant expression remaining only in skeletal and cardiac muscle 21^,^^22^. H19 acts through miR-675 to influence regeneration and differentiation 21^,^^22^^,^^23^. miR-15b was down-regulated in myasthenia gravis patients and was found to regulate IL-15 expression in a mouse model of the disease 8. It also stimulated cardiomyocyte apoptosis in response to ischaemia/reperfusion injury 24.

The highest number of differentially expressed miRNAs was found at four weeks of age and it is the only time point with down-regulated miRNAs. It is also the first time point with a differentially expressed muscle-enriched miRNA, i.e. miR-181a-1 and miR-181a-2, both of which are down-regulated at four weeks of age. Mir-181a was previously associated with the degree of muscle wasting following high-risk cardiothoracic surgery, with a high predictive value of 91%, despite some limitations in study design and low sensitivity (56%) 9. Apart from being a myomiR, miR-181a is also one of the mitochondria-associated miRNAs, also called mitomiRs. They bind to the mitochondrial outer membrane to regulate its metabolism, gene expression and function 25. Besides taking part in energy metabolism, mitochondria also have prominent roles in cell longevity and apoptosis. In line with this our group has previously shown that most differentially expressed proteins in *dy*^*3K*^/*dy*^*3K*^ (a severely affected LAMA2-CMD mouse model) muscle are coupled to energy and calcium metabolism/signalling 26. The most up-regulated miRNA at four weeks of age was miR-495, which is also up-regulated in various cardiac diseases, including cardiomyopathies associated with muscular dystrophies 27. Another differentially expressed miRNA involved in cardiopathy is miR-154, which is associated with increased fibrosis and reduced apoptosis 28. With an almost 6-fold increased expression in dystrophic muscle, miR-182 is involved in skeletal muscle atrophy 29, myocardial hypertrophy 30, muscle glucose utilisation 31 and the response to hormone replacement therapy in women 32. The most down-regulated miRNA at this time point was miR-155. It was reported to be involved in various processes in skeletal and cardiac muscle, such as pathological cardiac hypertrophy 33, skeletal muscle differentiation 34 and regeneration 35. Moreover, miR-155 regulates macrophage transition from a pro- to an anti-inflammatory state in skeletal muscle 35, which is an important step in muscle regeneration.

At six weeks of age myomiRs dominate the differentially expressed miRNA panel. It may suggest that compensatory mechanisms are at play and degeneration/regeneration cycles are intensified. Our lab has previously shown that miR-1, miR-133 and miR-206 are altered in quadriceps and plasma of *dy*^*2J*^*/dy*^*2J*^ and *dy*^*3K*^*/dy*^*3K*^ mice 6. Levels of miR-1 and miR-133 changed in opposite directions in muscle and plasma, i.e. both were down-regulated in quadriceps whilst up-regulated in blood plasma. MiR-206 was up-regulated in both muscle and plasma. MiR-1 and miR-133 are involved in differentiation and proliferation, respectively 31^,^^32^^,^^33^. MiR-206 on the other hand seems to be an important hub in gene networks in skeletal muscle given its involvement in fundamental processes such as muscle cell differentiation 32^,^^34^ and regeneration 35. Interestingly, work by Böttger et al. 36 presented evidence that the miR-206/133b cluster is in fact dispensable for skeletal muscle development and regeneration. MiR-486, a muscle-enriched miRNA, is also involved in various processes relevant for LAMA2-CMD. Hitachi et al. 37 showed that myostatin, a well-known negative regulator of muscle mass, acts via miR-486 to regulate the IGF-1/Akt/mTOR pathway; others have found similar results 38^,^^39^. MiR-486 also affects myoblast differentiation along with miR-206 34. One of the most interesting findings at six weeks of age is that miR-181a is up-regulated, given that it was down-regulated at four weeks. This makes it an interesting target for further investigation, coupled with its purported role in ageing, inflammation, and muscle and mitochondrial metabolism. Validating its targets in skeletal muscle would provide valuable insight into its function in this tissue.

Despite our interesting findings care must be taken when interpreting NGS results. NGS library preparation is known to induce biases that may favour certain sequences and thus compromise further analyses. For this reason, ideally, biomarkers should be validated with an orthogonal method, such as qPCR for example. We must also bear in mind that the clinical reality is quite different from a laboratory one. Our mice had standardised housing, diet, light-cycle, genetic background, etc., all of which differ amongst patients. Future work with clinical samples will have to deal with much higher data variability. Considering the low incidence of LAMA2-CMD it will be very difficult to run clinical trials with age- and sex-matched subjects. One of our goals was to match disease severity to miRNA profile. In the clinical setting this goal is likely to be hampered by the lack of standardised clinical outcomes for muscular dystrophies, another limitation in the field.

In summary, we were able to follow disease progression in LAMA2-CMD by analysing three distinct time points. Three-week-old *dy*^*2J*^*/dy*^*2J*^ muscle appears histologically normal with no functional deficit. Yet, CK is elevated and a few miRNAs are differentially expressed. At four weeks of age, muscles are histologically abnormal and show increased regeneration and functional decline (but no fibrosis). CK is increased and several differentially expressed miRNAs are detected. Finally, six-week-old *dy*^*2J*^*/dy*^*2J*^ muscle displays histological and functional impairment along with increased CK and the differentially expressed myomiRs. We would like to propose that miRNAs have the potential to distinguish disease stages and should be further investigated as biomarkers for LAMA2-CMD.

## Competing Interests

The authors declare that no competing interests exist.

## Data Availability

All data and access information are contained in the article.

## Corresponding Author

Bernardo Moreira Soares Oliveira: bernardo.moreira_soares_oliveira@med.lu.se

## Appendix

**Table:** **Table 1:** Differentially expressed miRNAs at the selected time points. Adjusted p < 0.05 and log2FoldChange > 1.

miRNA	log2FoldChange	p-adj
mmu-miR-675-3p	4.4063	0.0005	3wk
mmu-miR-675-5p	4.1857	0.0003	3wk
mmu-miR-1957a	2.9201	0.0003	3wk
mmu-miR-15b-5p	2.4532	0.0001	3wk
mmu-miR-320-5p	2.4024	0.0063	3wk
mmu-miR-495-3p	3.8713	0.0052	4wk
mmu-miR-369-3p	3.6786	0.0108	a4wk
mmu-miR-337-3p	3.6545	0.0028	4wk
mmu-miR-154-3p	3.6308	0.0028	4wk
mmu-miR-376b-3p	2.9790	0.0028	4wk
mmu-miR-182-5p	2.7268	0.0031	4wk
mmu-miR-127-3p	2.7131	0.0489	4wk
mmu-miR-148a-3p	2.5092	0.0028	4wk
mmu-miR-31-5p	2.1256	0.0012	4wk
mmu-miR-1839-5p	1.8521	0.0167	4wk
mmu-miR-21a-5p	1.3860	0.0108	4wk
mmu-miR-155-5p	-2.4041	0.0389	4wk
mmu-miR-615-3p	-2.1658	0.0028	4wk
mmu-miR-204-5p	-1.7558	0.0389	4wk
mmu-miR-187-3p	-1.7246	0.0477	4wk
mmu-miR-181a-1-5p	-1.3911	0.0389	4wk
mmu-miR-181a-2-5p	-1.3911	0.0389	4wk
mmu-miR-378c	-1.1433	0.0389	4wk
mmu-miR-486a-5p	4.7623	0.0002	6wk
mmu-miR-486b-5p	4.7524	0.0002	6wk
mmu-miR-5108	4.5049	0.0076	6wk
mmu-miR-206-3p	4.1917	0.0031	6wk
mmu-miR-8101	4.0483	0.0127	6wk
mmu-miR-675-5p	3.9570	0.0031	6wk
mmu-miR-133b-3p	3.7315	0.0221	6wk
mmu-miR-1a-2-3p	3.5710	0.0103	6wk
mmu-miR-1a-1-3p	3.5710	0.0103	6wk
mmu-miR-133a-1-3p	2.8598	0.0103	6wk
mmu-miR-133a-2-3p	2.8598	0.0103	6wk
mmu-miR-1957a	2.5676	0.0401	6wk
mmu-miR-5100	1.9848	0.0127	6wk
mmu-miR-7a-2-5p	1.7627	0.0204	6wk
mmu-miR-7a-1-5p	1.7603	0.0204	6wk
mmu-miR-181a-1-5p	1.5137	0.0190	6wk
mmu-miR-181a-2-5p	1.5137	0.0190	6wk

**Supplemental Figures**

**Figure:**
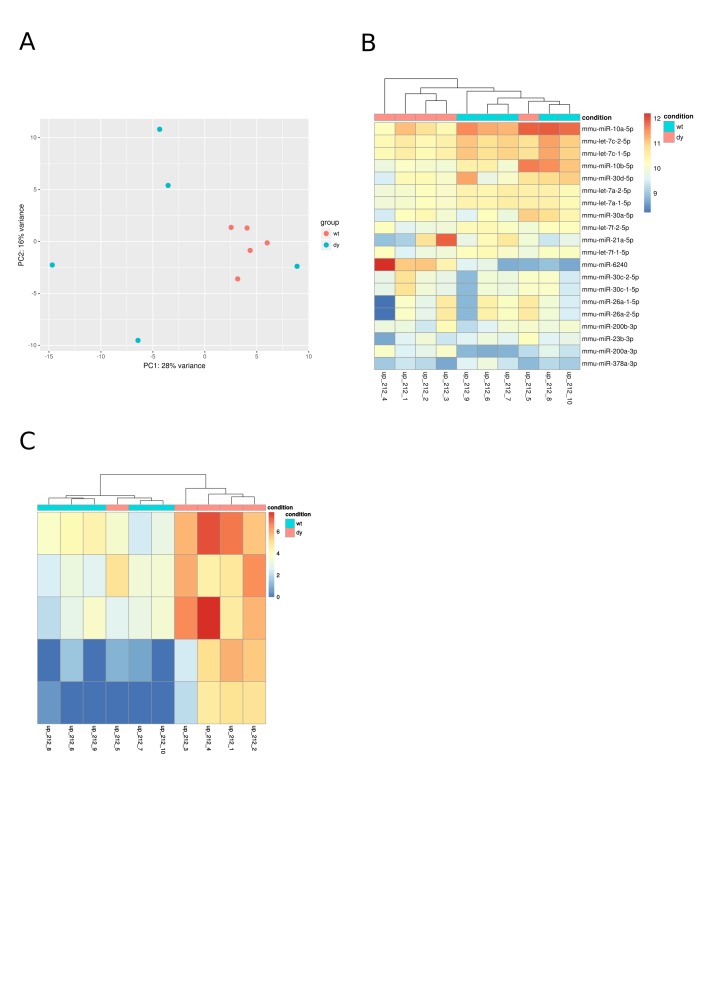
**Supplemental Fig. 1:** Diagnostic plots: principal component analysis (PCA) shows how well samples group based on global gene expression; heatmaps are color-coded values from an expression matrix, which may or may not include all the genes. A: PCA of three-week-old samples; B: Heatmap of the 20 mostly expressed miRNAs at three weeks of age; C: Heatmap of differentially expressed miRNAs at three weeks of age.

**Figure:**
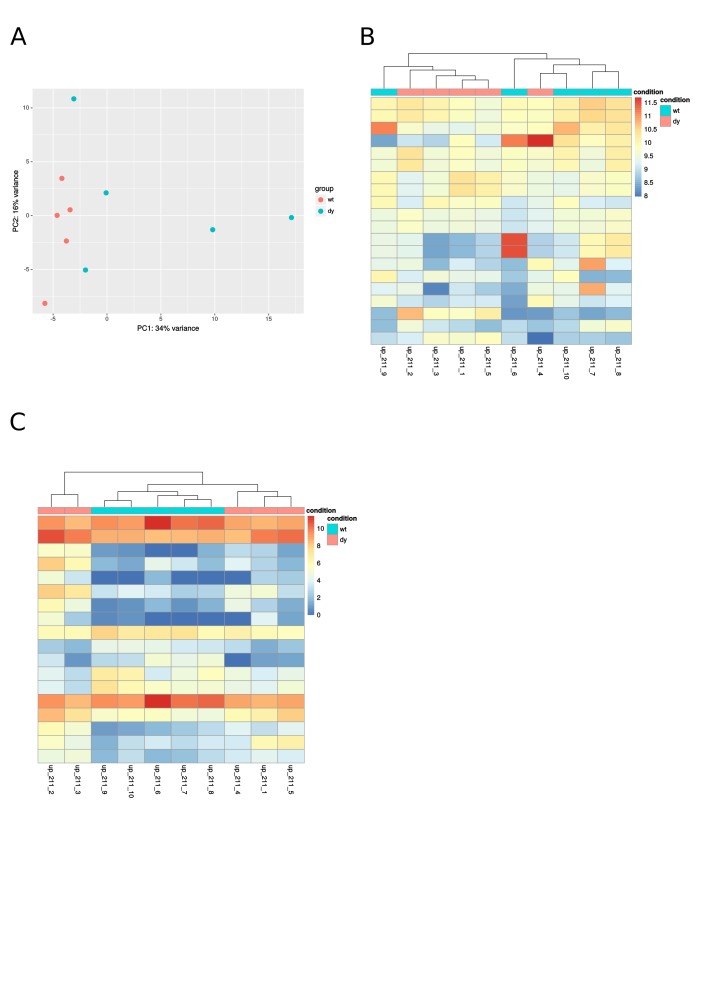
**Supplemental Fig. 2:** A: PCA of four-week-old samples; B: Heatmap of the 20 mostly expressed miRNAs at four weeks of age; C: Heatmap of differentially expressed miRNAs at four weeks of age.

**Figure:**
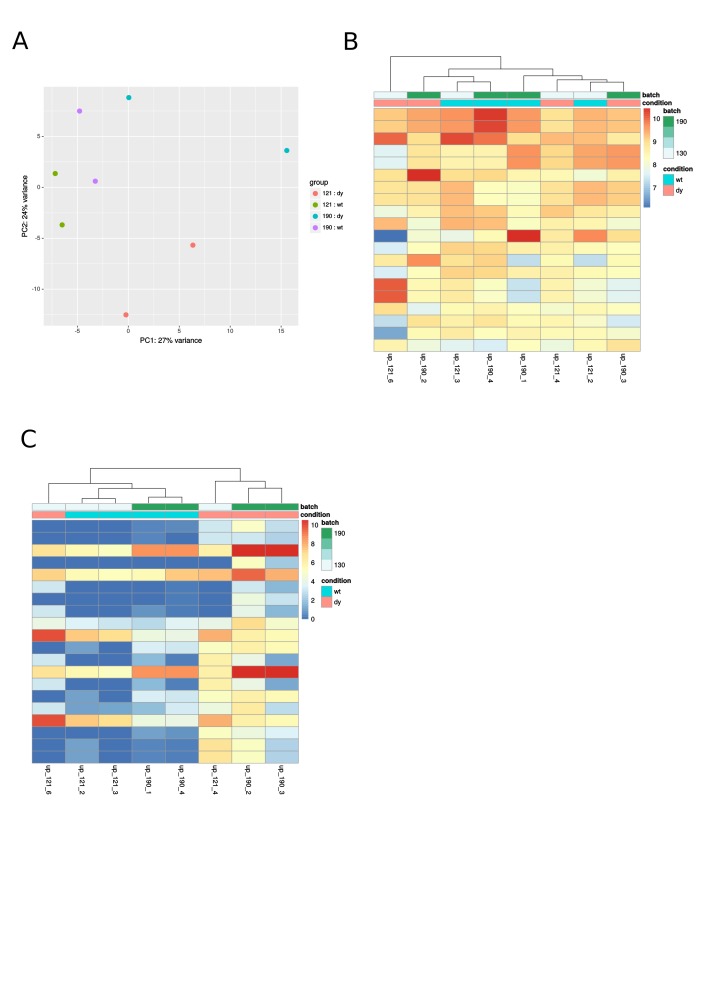
**Supplemental Fig. 3:** A: PCA of six-week-old samples; B: Heatmap of the 20 mostly expressed miRNAs at six weeks of age; C: Heatmap of differentially expressed miRNAs at six weeks of age.
